# Enriched sensory feedback delivered during a voluntary action boosts subjective time compression

**DOI:** 10.3389/fpsyg.2023.1140569

**Published:** 2023-08-10

**Authors:** Sayako Ueda, Shingo Shimoda

**Affiliations:** ^1^Department of Psychology, Japan Women’s University, Tokyo, Japan; ^2^Graduate School of Medicine, Nagoya University, Nagoya, Japan; ^3^Center for Brain Science, RIKEN, Wako, Japan

**Keywords:** the sense of time, the sense of agency, sensory feedback enrichment, duration reproduction, continuous action

## Abstract

**Introduction:**

The subjective experience of time can be influenced by various factors including voluntary actions. In our previous study, we found that the subjective time experience of an action outcome can be compressed when an individual performs a continuous action compared to a single action, suggesting that the sense of agency (SoA), the feeling of control over one’s own action outcomes, contributes to the subjective time compression. We hypothesized that enhancing SoA by providing sensory feedback to participants would further compress the subjective time experience.

**Methods:**

To test the hypothesis, we used a temporal reproduction task where participants reproduced the duration of a previously exposed auditory stimulus by performing different voluntary actions: a combination of single actions with single auditory feedback, continuous action with single auditory feedback, or continuous action with multiple auditory feedback.

**Results:**

The results showed that the continuous action conditions, regardless of the type of auditory feedback, led to a compression of the subjective time experience of the reproduced tone, whereas the single action condition did not. Furthermore, a greater degree of subjective time compression during continuous action and a stronger SoA were revealed when enriched with multiple auditory feedback.

**Discussion:**

These results indicate that enriching auditory feedback can increase subjective time compression during voluntary action, which in turn enhances SoA over action outcomes. This suggests the potential for developing new techniques to artificially compress the subjective time experience of daily events.

## Introduction

In everyday life, people formulate intentions to act and then move their bodies to produce a desired outcome in the external environment. Such voluntary actions can compress the subjective time experience of their outcome, irrespective of sensory modality, thereby facilitating optimized interactions with the external environment (Haggard et al., 2002; Engbert et al., 2007, 2008; Humphreys and Buehner, 2009; Imaizumi et al., 2019). The intentional binding effect is a well-known example, where the perceived interval between a voluntary action and its outcome is compressed. Haggard et al. (2002) first introduced this effect by asking participants to report the onset of a single voluntary action (a key press) and of a subsequent sensory event (a tone). They found that voluntary keypresses, but not involuntary ones induced by transcranial magnetic stimulation (TMS), were perceived as shifted in time toward the subsequent tones, and vice versa, compared to control conditions. In addition, voluntary actions can also subjectively compress the temporal duration of action outcomes. Imaizumi et al. (2019) showed that, compared to cases where the experimenter moved the participant’s finger so that keypresses were performed involuntarily, the duration of a visual event that began and ended according to a participant’s voluntary keypress was subjectively estimated to be shorter. Since the compression of subjective time experience should reflect a mental operation to bind in time pairs of events that can plausibly be linked by self-causation, such subjective time compression is thought to play a role in maintaining the experience of controlling action outcomes in the external environment, which is commonly referred to as the sense of agency (Haggard and Chambon, 2012; Haggard, 2017).

The internal comparator model (Blakemore et al., 1999; Frith et al., 2000) is a well-established theoretical framework that has been supported by a substantial body of research using various paradigms and measures (Sato and Yasuda, 2005; Ebert and Wegner, 2010; Kühn et al., 2011; Farrer et al., 2013; Hon et al., 2013; Kawabe, 2013; Wen et al., 2015). According to this model, the sense of agency arises from the match between voluntary actions and their outcomes. Specifically, when it comes to voluntary motor control, the motor system’s internal forward model uses an efference copy of the motor command to predict the action’s outcome before it is executed (Wolpert et al., 1995; Wolpert and Ghahramani, 2000; Wolpert and Flanagan, 2001). For example, when turning on a light switch, the brain sends a motor command to press the switch, and the efference copy of this command is used to predict the resulting hand movement and the light’s outcome. The predicted outcome is then compared to the actual outcome, and the difference between the two is known as the prediction error. If the predicted and actual outcomes match (i.e., the prediction error is zero), the person is more likely to experience the sense of agency over the outcome. Conversely, as the prediction error increases, individuals become less able to attribute the outcome to their own actions, leading to the reduced or absent sense of agency. This idea is supported by a wealth of experimental studies, such as those in which participants judge whether a video they are watching shows their own or someone else’s hand movements. When presented with visual feedback of someone else’s hand and there is a mismatch between the predicted and actual outcome, participants are less able to attribute the movements to their own actions (Daprati et al., 1997; Sirigu et al., 1999; Van Den Bos and Jeannerod, 2002; Tsakiris et al., 2005). Similarly, when a computer alters the visual feedback to create a mismatch between predicted and actual outcomes, participants are less willing to attribute the feedback to their own actions (Ueda et al., 2021).

To increase in the number of matches between predicted and actual action outcomes during a voluntary action may be a possible way to increase the compression of the subjective time experience of the action outcome, leading to an enhanced sense of agency. In a recent study, we investigated how the subjective time experience of action outcomes changes depending on the type of voluntary action. Specifically, we conducted a temporal reproduction task in which participants reproduced the duration of a previously exposed auditory stimulus using different types of voluntary actions (Ueda and Shimoda, 2021). We compared the subjective time experience of a combination of single actions (in which the reproduction tone started and ended in response to key presses) with that of a continuous action (in which the reproduction tone was produced by continuously turning a steering wheel). We found that the compression of the subjective time experience of the reproduction tone was greater for the continuous action than for the combination of single actions. This effect could not be explained by increased physical or mental workload. Our findings suggest that performing the continuous action provided participants with more opportunities to create matches between predicted and actual outcomes than performing a single action. During the continuous action, participants may have constantly predicted action outcomes and compared them with actual outcomes, accumulating more than two matches. In contrast, during the single action, participants had a maximum of two opportunities to predict action outcomes (i.e., at the beginning and at the end of the sound reproduction by key press). While our previous study suggested that increasing the number of matches between predicted and actual action outcomes during a voluntary action can artificially compress the subjective time experience of the action outcome, we did not manipulate the opportunities to create such matches across different conditions of the same voluntary action type. Therefore, to adequately test the extent to which an increase in the number of matches between predicted and actual outcomes enhances subjective time compression and the sense of agency over the action outcome, it is necessary to manipulate the number of opportunities to create such matches across conditions of the same voluntary action type.

Enriching sensory feedback during voluntary actions by increasing the number of feedback change points may provide additional opportunities to generate matches between predicted and actual outcomes. This, in turn, may enhance individuals to predict their action outcomes, compare them to actual outcomes, and accumulate matches. Building on this idea, the current study sought to investigate how the change in sensory feedback provided during a voluntary action affects the compression of subjective time and the sense of agency over the action outcome. We used a temporal reproduction task similar to our previous study (Ueda and Shimoda, 2021) where participants continuously turned a steering wheel to reproduce the duration of an auditory stimulus (a tone) they were previously exposed to. In one condition, a harmony tone consisting of six different tones was constantly presented as the reproduction tone (the single auditory feedback condition). In the other condition, the six different tones were presented independently depending on the position of the steering wheel while it was continuously turned (the multiple auditory feedback condition). This manipulation allowed us to vary the richness of sensory feedback, i.e., the number of feedback change points, in relation to action speed. If the subjective time experience of the reproduction tone was more compressed in the multiple auditory feedback condition than in the single auditory feedback condition, this would suggest that enriching sensory feedback during a voluntary action increases the number of matches between predicted and actual outcomes, leading to an increase in subjective time compression. In addition, to investigate the sense of agency, we asked participants to directly judge their own notion of control during the task, which is a common way of quantifying the sense of agency (Haggard, 2017). If self-reported ratings of control were higher in the multiple auditory feedback condition compared to the single auditory feedback condition, this would suggest that enriching sensory feedback during a voluntary action increases the sense of agency over the action outcome by increasing the number of matches between predicted and actual outcomes.

## Methods

### Participants

A total of 20 experimentally naïve adults (13 women and 7 men) aged 20–39 years (*M* = 29.3 years; SD = 6.7 years) participated in this study and received monetary compensation for their involvement. The sample size was determined through a power analysis using G*Power (Faul et al., 2007) and the solution proposed by David Morse (Shirai and Ogawa, 2020).^1^ The analysis indicated that at least 17 participants were needed to detect a medium effect (*f* = 0.25) with 80% power and a significance level of 0.05 for a two-way repeated measures within-subjects analysis of variance (ANOVA). All participants had normal or corrected-to-normal vision and were right-handed as determined by the Edinburgh Handedness Inventory (Oldfield, 1971). Participants were also screened for normal visuomotor function using the Grooved Pegboard Test (Lafayette Instruments, Lafayette, IN). Written informed consent was obtained from all participants in accordance with the study protocol approved by the RIKEN Research Ethics Committee [Wako3 30–13(2)]. All procedures were conducted in accordance with applicable guidelines and regulations.

### Apparatus, setup, and procedure

**Figure 1:**
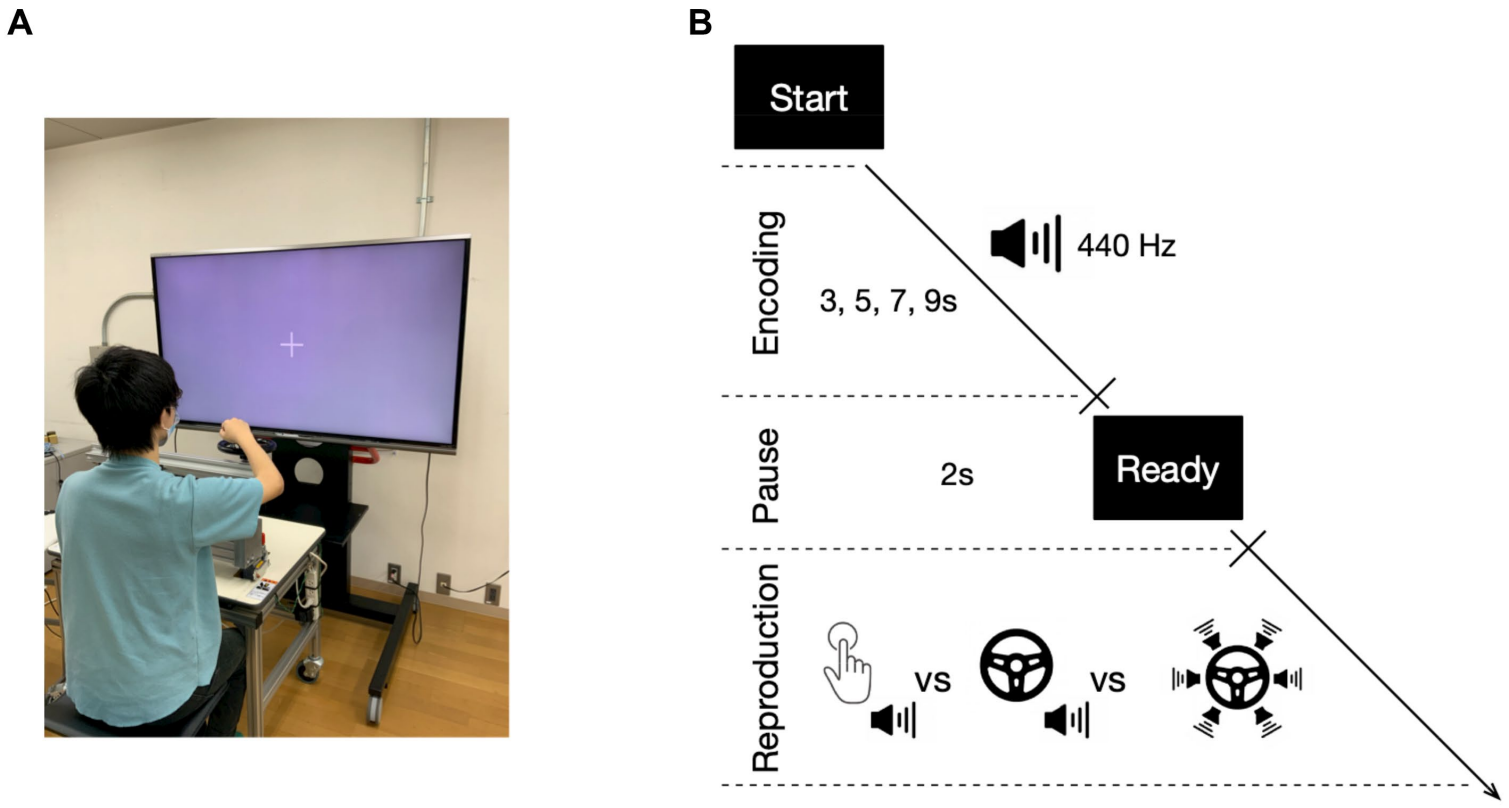
Experimental apparatus and temporal reproduction task. Participants were seated in front of a table with a keypad and a steering wheel, positioned in front of a monitor **(A)**. During the temporal reproduction task **(B)**, participants were presented with tones of varying durations and asked to reproduce the duration by either pressing a key twice with a single auditory feedback (the single action with single auditory feedback condition: single-single), turning the steering wheel with a single auditory feedback (the continuous action with single auditory feedback condition: continuous-single), or turning the steering wheel with multiple auditory feedback (the continuous action with multiple auditory feedback condition: continuous-multiple).

Figure 1 displays the apparatus and task design utilized in the experiment, which was conducted in a dimly lit room (see also Ueda and Shimoda, 2021). Participants were seated at a distance of 60 cm from a 60-inch LCD monitor (LC60XL10, SHARP Corp., Japan). A keypad (BSTK10, BUFFALO Inc., Japan) and a steering wheel (diameter of 20 cm, see also Itkonen et al., 2019) were placed on a table in front of them (Figure 1A: The volunteer provided informed consent regarding the inclusion of their face in the figure for publication). In the temporal reproduction task, participants were instructed to reproduce the duration of a previously presented tone (with a duration of 3, 5, 7, or 9-s) by pressing the key or turning the steering wheel. Each trial began with a “Start” prompt presented on the monitor, followed immediately by the encoding phase, signaled by a fixation cross presented on the screen after the participant pressed the key with their right hand. In this phase, a 440 Hz tone was presented for one of four durations (the encoding tone). When the tone ended, the fixation cross disappeared, and a “Ready” prompt was presented for 2-s. This was followed by the reproduction phase, in which the fixation cross was presented again, and the participant was expected to create the reproduction tone (Figure 1B). Two different action types were utilized to create the reproduction tone. In the first action type, the reproduction tone was generated by pressing the key twice: first to start the tone and then again to end the tone, indicating that the duration of the encoding tone had elapsed (single action). In the second action type, the participant produced the reproduction tone by continuously turning the steering wheel outward until they estimated that the duration of the encoding tone had elapsed (continuous action). Two different types of reproduction tone were presented. The first was a harmony tone consisting of six different tones (520, 590, 660, 700, 780, and 880 Hz), presented in both the single action with single auditory feedback condition (single-single) and continuous action with single auditory feedback condition (continuous-single). These two conditions were similar to those in Experiment 1 of a previous study by Ueda and Shimoda (2021), except that a different reproduction tone was used (640 Hz in the previous study). The other type of reproduction tone was only used in the continuous action, where the 360-degree steering wheel was divided into six 60-degree areas, each with a different tone (520, 590, 660, 700, 780, and 880 Hz). The six different tones were independently presented depending on the handle position of the steering wheel, resulting in a variable reproduction tone based on the speed of the continuous action (continuous-multiple). Participants were instructed to turn the steering wheel at a comfortable constant speed without using methods such as counting or tapping to complete the task. Each participant’s comfortable constant speed was determined prior to the experiment. During the experiment, the position of the steering wheel was recorded at 1/60 s intervals using a computer-controlled system. Stimuli presentation and steering wheel position recording were both controlled by MATLAB software with the Psychtoolbox extension (Brainard, 1997). The participants were randomly assigned to one of six different patterns, which determined the order of the task conditions (i.e., the single-single condition, the continuous-single condition, and the continuous-multiple condition). This ensured counter-balancing of condition order across participants. Before each task condition, participants completed five practice trials with an encoding duration of 2 s to familiarize themselves with the task. For each task condition, participants completed 24 trials (experimental session), with six trials for each tone duration (3, 5, 7, and 9-s), presented in random order. Participants were given a 1-min break between the practice and experimental sessions, and on average, the experiment lasted 35 min. After the experiment, participants rated their sense of agency, i.e., the degree to which they felt that the reproduction tone was under their control, for each condition using a seven-point scale (1 = not at all; 7 = a lot). This allowed us to evaluate the participants’ explicit sense of agency over the reproduction tone.

### Data analysis

Temporal reproduction performance was evaluated for each trial using the temporal reproduction error, calculated as the reproduced duration divided by the encoded duration (Brown, 1985; Brown, 1997; Glicksohn and Hadad, 2012; Ueda and Shimoda, 2021). An error value of 1 indicated accurate reproduction of the encoded duration, whereas values greater than 1 indicated over-reproduction and values less than 1 indicated under-reproduction. The internal clock model (Creelman, 1962; Treisman, 1963; Church, 1984; Gibbon et al., 1997) proposes a pacemaker that generates ticks to be accumulated by the accumulator, and over-reproduction and under-reproduction can be explained by changes in the tick rate of the internal clock. Specifically, slowing of the tick rate leads to over-reproduction (subjective time compression) because of the accumulation of fewer ticks, while accelerating of the tick rate leads to under-reproduction (subjective time dilation) due to the accumulation of more ticks. Therefore, we used error values greater than 1 as an index of subjective time compression and values less than 1 as an index of subjective time dilation for the reproduction tone. In the continuous-single and continuous-multiple conditions, we also assessed continuous action performance for each trial using the speed of action, calculated as the mean steering wheel velocity during the reproduction phase. The relationship between continuous action performance and temporal reproduction performance was further explored.

The temporal reproduction error was tested using a one-sample t-test to compare the values to 1, allowing us to determine whether the reproduced duration was compressed. We then conducted a two-way repeated measures ANOVA with task condition (single-single, continuous-single, and continuous-multiple) and duration condition (3, 5, 7, and 9-s) as repeated measures factors to compare the task conditions. When we found a significant interaction between task and duration condition, we performed subsequent simple main effect analyses. When significant simple main effects of task or duration conditions were found, we conducted multiple comparisons. We corrected *p*-values using Shaffer’s modified sequentially rejective Bonferroni procedure (Shaffer, 1986). If the interaction was not significant, but significant simple main effects of task or duration conditions were found, we performed subsequent multiple comparisons using Shaffer’s modified sequentially rejective Bonferroni procedure (Shaffer, 1986). To examine the relationship between the temporal reproduction error and the mean steering wheel velocity for each duration condition, we calculated Pearson’s correlation coefficients and conducted a one-sample *t*-test to compare the mean correlation coefficients with a value of 0. We also conducted a paired *t*-test to compare the mean correlation coefficients between the continuous-single and continuous-multiple conditions. The self-reported ratings of control were analyzed using a one-way repeated measures ANOVA of the task condition (single-single, continuous-single, and continuous-multiple). If we found a significant effect of the task condition, we performed subsequent multiple comparisons using Shaffer’s modified sequentially rejective Bonferroni procedure (Shaffer, 1986). Finally, to examine the relationship between the subjective time compression and the explicit rating of the sense of agency, we calculated Pearson’s correlation coefficients using the mean temporal reproduction error in the duration conditions for each task condition and the self-reported rating of control. We set the significance threshold at *p* < 0.05 for all tests. We performed statistical analyses using R software (version 3.3.2 for Mac).

## Results

**Figure 2:**
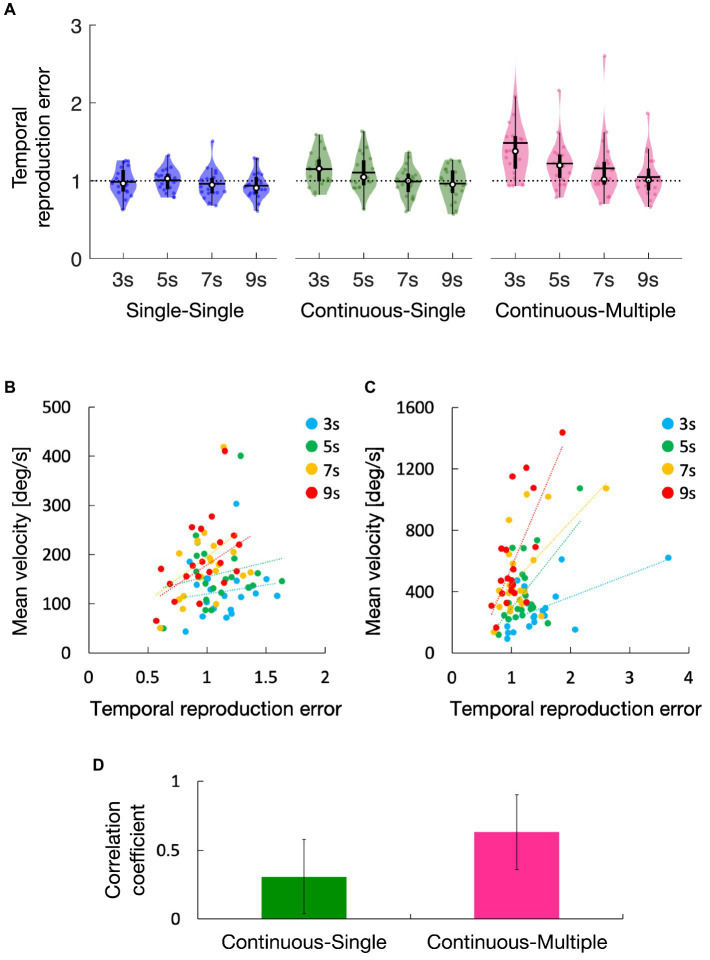
Temporal reproduction error for the single-single, continuous-single, and continuous-multiple conditions **(A)**. Regression lines were fitted to the temporal reproduction error and steering wheel velocity for each duration condition in the continuous-single condition **(B)** and the continuous-multiple condition **(C)**, and their average correlation coefficients were calculated **(D)**. The white circles within the violin plots represent the median; the solid horizontal lines give the mean **(A)**. Error bars within the bar graph represent the 95% confidence intervals **(D)**. Statistical analyses revealed that, compared to 1 (dotted line), the temporal reproduction errors were higher in the 3-s condition in the continuous-single condition and higher in the 3-s and 5-s, conditions in the continuous-multiple condition, but there were no significant differences for all duration conditions in the single-single condition **(A)**. The slopes of the lines were positive in both conditions **(B,C)**, but they were more strongly positive in the continuous-multiple condition **(C)** than in the continuous-single condition **(B)**, and the average correlation coefficient was significantly higher in the continuous-multiple condition than in the continuous-single condition **(D)**.

The average temporal reproduction error for the single-single, continuous-single, and continuous-multiple conditions are shown in Figure 2A. In the single-single condition, the temporal reproduction errors seemed lower than 1, while those in the continuous-single and continuous-multiple conditions seemed higher than 1, particularly in the 3-s and 5-s conditions for the continuous-single condition, and in all of the duration conditions for the continuous-multiple condition. A one-sample *t*-test showed that the temporal reproduction errors in the single-single condition were not significantly different among the duration conditions compared with a value of 1 (3 s: *t*[19] = 0.33, *p* = 0.74, *d* = 0.07, 5 s: *t*[19] = 0.19, *p* = 0.85, *d* = 0.04, 7 s: *t*[19] = 0.93, *p* = 0.36, *d* = 0.21, 9 s: *t*[19] = 1.63, *p* = 0.12, *d* = 0.36). On the other hand, in the continuous-single condition, the temporal reproduction errors were significantly higher in the 3-s condition (*t*[19] = 3.17, *p* < 0.01, *d* = 0.71), but not significantly different in the 5-s condition (*t*[19] = 2.04, *p* = 0.06, *d* = 0.46), 7-s condition (*t*[19] = 0.19, *p* = 0.85, *d* = 0.04), and 9-s condition (*t*[19] = 0.78, *p* = 0.45, *d* = 0.17). The temporal reproduction errors in the continuous-multiple condition were significantly higher in the 3-s condition (*t*[19] = 3.62, *p* < 0.01, *d* = 0.81) and 5-s condition (*t*[19] = 3.35, *p* < 0.01, *d* = 0.75), but not significantly different in the 7-s condition (*t*[19] = 1.73, *p* = 0.09, *d* = 0.39) and 9-s condition (*t*[19] = 0.79, *p* = 0.43, *d* = 0.18).

The temporal reproduction errors in Figure 2A seemed highest in the continuous-multiple condition and lowest in the single-single condition (i.e., continuous-multiple > continuous-single > single-single). All three task conditions showed a similar trend, i.e., the temporal reproduction errors decreased as the duration lengths increased. This trend appeared to be particularly strong in the continuous-multiple condition. A two-way repeated measures ANOVA showed main effects of task condition [*F*(2, 38) = 7.77, *p* < 0.01, *ηp*^2^ = 0.29] and duration condition [*F*(3, 57) = 18.64, *p* < 0.01, *ηp*^2^ = 0.50], as well as a significant interaction between the task and duration condition [*F*(6, 114) = 6.80, *p* < 0.01, *ηp*^2^ = 0.26]. We then performed subsequent simple main effect analyses and found the expected simple main effects of task condition in the 3-s, 5-s, and 7-s conditions [3 s: *F*(2, 38) = 10.35, *p* < 0.01, *ηp*^2^ = 0.35, 5 s: *F*(2, 38) = 7.12, *p* < 0.01, *ηp*^2^ = 0.27, 7 s: *F*(2, 38) = 4.44, *p* = 0.02, *ηp*^2^ = 0.19], but not in the 9-s condition [*F*(2, 38) = 2.05, *p* = 0.14, *ηp*^2^ = 0.10]. Subsequent multiple comparisons for the significant simple main effect of the task condition in each duration condition revealed that temporal reproduction errors were significantly greater in the continuous-multiple condition compared with the single-single condition in the 3-s, 5-s, and 7-s conditions (3 s: *t*[19] = 3.65, *p* < 0.01, *d* = 0.82, 5 s: *t*[19] = 3.27, *p* = 0.01, *d* = 0.73, 7 s: *t*[19] = 2.82, *p* = 0.03, *d* = 0.63), significantly greater in the continuous-single condition compared with the single-single condition in the 3-s and 5-s conditions (3 s: *t*[19] = 4.80, *p* < 0.01, *d* = 1.07, 5 s: *t*[19] = 2.47, *p* = 0.02, *d* = 0.55) but not the 7-s condition (*t*[19] = 0.62, *p* = 0.54, *d* = 0.14), significantly greater in the continuous-multiple condition compared with the continuous-single condition in the 3-s condition (*t*[19] = 2.52, *p* = 0.02, *d* = 0.56) but not the 5-s and 7-s conditions (5 s: *t*[19] = 1.87, *p* = 0.08, *d* = 0.42, 7 s: *t*[19] = 1.86, *p* = 0.08, *d* = 0.42). We also found the expected simple main effects of duration condition in the continuous-single and continuous-multiple conditions [continuous-single: *F*(3, 57) = 9.30, *p* < 0.01, *ηp*^2^ = 0.33, continuous-multiple: *F*(3, 57) = 17.42, *p* < 0.01, *ηp*^2^ = 0.48] but not in the single-single condition [*F*(3, 57) = 1.01, *p* = 0.39, *ηp*^2^ = 0.05]. Subsequent multiple comparisons for the significant simple main effect of the duration condition in each task condition revealed that in the continuous-single condition, the temporal reproduction error was significantly greater in the 3-s condition compared with the 7-s and 9-s conditions (7 s: *t*[19] = 3.70, *p* < 0.01, *d* = 0.83, 9 s: *t*[19] = 4.15, *p* < 0.01, *d* = 0.93) and significantly greater in the 5-s condition compared with the 7-s and 9-s conditions (7 s: *t*[19] = 2.81, *p* = 0.03, *d* = 0.63, 9 s: *t*[19] = 2.87, *p* = 0.03, *d* = 0.64), but not significantly different between the 3-s and 5-s conditions (*t*[19] = 1.36, *p* = 0.38, *d* = 0.31) or between the 7-s and 9-s conditions (*t*[19] = 0.78, *p* = 0.44, *d* = 0.18). In the continuous-multiple condition, the temporal reproduction error was significantly greater in the 3-s condition compared with the 5-s, 7-s, and 9-s conditions (5 s: *t*[19] = 3.38, *p* < 0.01, *d* = 0.76, 7 s: *t*[19] = 5.34, *p* < 0.01, *d* = 1.07, 9 s: *t*[19] = 4.81, *p* < 0.01, *d* = 1.07), significantly greater in the 5-s compared with the 9-s condition (*t*[19] = 4.52, *p* < 0.01, *d* = 1.01), and significantly greater in the 7-s compared with the 9-s condition (*t*[19] = 2.46, *p* = 0.04, *d* = 0.55). However, we found no significant difference between the 5-s and 7-s conditions (*t*[19] = 1.36, *p* = 0.19, *d* = 0.30). The results suggest that the reproduction of the encoded duration was prolonged in both the continuous-single and continuous-multiple conditions, indicating compression of the subjective time of the reproduction tone. Furthermore, the amount of compression was greater in the continuous-multiple condition compared to the continuous-single condition. However, as the duration increased, the amount of compression decreased until it approached an accurate value. This trend was more pronounced in the continuous-multiple condition than in the continuous-single condition. On the other hand, in the single-single condition, the encoded duration was accurately reproduced regardless of the duration, without any significant compression of the subjective time of the reproduction tone.

Figures 2B,C show the regression lines fitted to reproduction time error and steering wheel velocity for each duration condition (the continuous-single condition: B, the continuous-multiple condition: C). Although the slopes of the lines were positive in both conditions, they seemed more strongly positive in the continuous-multiple condition than in the continuous-single condition. As shown in Figure 2D, a one-sample t-test revealed that the correlation coefficients between these metrics were significantly higher than 0 in the continuous-single condition (*t*[3] = 3.61, *p* = 0.04, *d* = 1.81) and the continuous-multiple condition (*t*[3] = 21.32, *p* < 0.01, *d* = 10.66). A paired *t*-test revealed that the correlation coefficients were significantly higher in the continuous-multiple condition than in the continuous-single condition (*t*[3] = 5.07, *p* = 0.01, *d* = 2.53). The results suggest that there was a positive relationship between the speed of action and the subjective time compression in both conditions, and that the relationship was stronger in the continuous-multiple condition than in the continuous-single condition.

**Figure 3:**
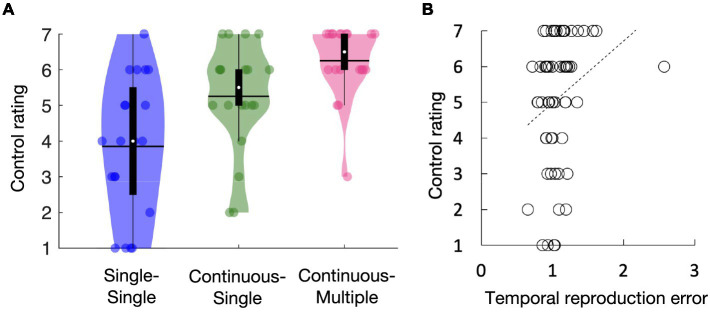
Self-reported ratings of control for the single-single, continuous-single, and continuous-multiple conditions **(A)**. Regression line fitted to the mean temporal reproduction error in the duration conditions for each task condition and the self-reported ratings of control **(B)**. The white circles within the violin plots represent the median; the solid horizontal lines give the mean **(A)**. Statistical comparisons revealed that the self-reported ratings of control were highest in the continuous-multiple condition, followed by the continuous-single condition, and lowest in the single-single condition **(A)**. The slope of the regression line was positive, indicating a positive relationship between the self-reported ratings of control and the temporal reproduction error **(B)**.

Figure 3A shows the average self-reported rating of control for the single-single, continuous-single, and continuous-multiple conditions. The self-reported ratings of control seemed highest in the continuous-multiple condition and lowest in the single-single condition (i.e., continuous-multiple > continuous-single > single-single). A one-way repeated measures ANOVA showed main effects of task condition [*F*(2, 38) = 12.29, *p* < 0.01, *ηp*^2^ = 0.39]. Subsequent multiple comparisons revealed that the self-reported ratings of control were significantly higher in the continuous-multiple condition than in the single-single and continuous-single conditions (*t*[19] = 5.02, *p* < 0.01, *d* = 1.12, *t*[19] = 3.08, *p* < 0.01, *d* = 0.69, respectively) and significantly higher in the continuous-single condition than in the single-single condition (*t*[19] = 2.28, *p* = 0.03, *d* = 0.51). Figure 3B shows the regression line fitted to the mean temporal reproduction error for the duration conditions for each task condition and the self-reported rating of control, which had a positive slope. Pearson’s correlation analysis revealed a significant positive relationship between the temporal reproduction error and the self-reported ratings of control (*r* = 0.26, *p* = 0.04). The results suggest that the explicit rating of the sense of agency was higher in the continuous action condition than in the single action condition. Moreover, this trend was more pronounced when the continuous action was accompanied by auditory feedback containing multiple tones. We also observed a positive correlation between the amount of subjective time compression and the explicit ratings of the sense of agency.

In summary, our study found that the continuous action led to a compression of the subjective time of the reproduction tone presented as the action outcome, regardless of the type of auditory feedback, while the single action did not. Additionally, we observed that explicit ratings of the sense of agency were stronger for the continuous action than for the single action. We also found that the subjective time compression caused by the continuous action decreased as the encoded duration length increased, leading to more accurate estimations. The speed of the continuous action was positively correlated with the amount of subjective time compression. These trends were more pronounced when the continuous action was accompanied by enriched auditory feedback. Finally, a positive relationship was observed between the subjective time compression and explicit ratings of the sense of agency.

## Discussion

Regardless of the sensory modality, voluntary actions can compress the perceived duration of their outcomes, which helps to maintain the experience of control over external events known as the sense of agency. Recent research suggests that when performing a voluntary action, increasing the number of matches between predicted and actual action outcomes can intensify the compression of the subjective time experience of the action outcome and enhance the sense of agency. However, it remains unclear whether increasing the number of opportunities to create such matches between predicted and actual outcomes can further modulate the compression of subjective time experience. Such an investigation could have implications for the development of new techniques for artificially altering our time experiences of daily events involving continuous actions, such as driving.

In this study, we aimed to investigate how the subjective experience of time for action outcomes is influenced by the change in sensory feedback provided. To achieve this, we conducted a temporal reproduction task, in which participants were asked to reproduce the duration of an auditory stimulus that they had encountered during different conditions: a combination of single actions with single auditory feedback, continuous action with single auditory feedback, or continuous action with multiple auditory feedback. As a result, consistent with our previous findings (Ueda and Shimoda, 2021), our results showed that the continuous action conditions, regardless of the type of auditory feedback, led to a compression of the subjective time experience of the reproduced tone, whereas the single action condition did not. Furthermore, we found a positive correlation between the degree of time compression and the speed of the continuous action. It is important to note that this compression of subjective time experience observed during the continuous action cannot be explained by an increase in physical or mental workload. This is because we would expect the physical or mental workload to be higher during the continuous action than during the single action, and for it to increase as the speed of the continuous action increases. Recent meta-analyses have shown that higher levels of physical or mental workload usually lead to subjective time dilation (Block et al., 2010, 2016), which is the opposite pattern of what we observed in this study. Therefore, our results suggest that the observed compression of subjective time experience during continuous action is not caused by the increased workload.

Moreover, our study revealed a more significant compression of subjective time experience during the continuous action when enriched with multiple auditory feedback. This could be attributed to the increased richness of the auditory feedback, which may have diverted attention away from the reproduction tone. Prior research suggests that when attentional resources are divided, attention to an event decreases, and the perception of the event’s duration is compressed (Coull et al., 2004). Thus, the reproduction tone may have disrupted participants’ attention, and the faster pace of the continuous action may have required more attentional resources, resulting in a greater lack of attention to the reproduction tone and a subsequent increase in subjective time compression. However, our findings on the sense of agency contradict this explanation. In fact, explicit ratings of the sense of agency were higher when the continuous action was accompanied by multiple auditory feedback, and subjective time compression was positively correlated with explicit ratings of agency. Previous studies that measured the sense of agency during the primary task, along with a secondary task that required attentional resources, found that explicit ratings of the sense of agency over the primary task decreased as the attentional demands of the secondary task increased (Hon et al., 2013; Wen et al., 2016; Hon, 2017). This suggests that generating a sense of agency requires attention. The effect of decreased attention on explicit ratings of the sense of agency appears to be opposite to what we observed in this study. Therefore, based on our findings, it seems that the observed increase in the compression of subjective time during the continuous action with multiple auditory feedback cannot be attributed to a lack of attention to the reproduction tone caused by the enriched auditory feedback. Rather, the fact that there was a positive correlation between the subjective time compression and explicit ratings of the sense of agency suggests that the enriched auditory feedback during the continuous action could increase the number of matches between predicted and actual outcomes, which may enhance the compression of the subjective time experience of the action outcome and the associated sense of agency. We can therefore conclude that the increased richness of auditory feedback enhanced the alignment between predicted and actual outcomes, resulting in a stronger sense of agency and a more pronounced compression of the subjective time experience of the action outcome. These findings suggest that enriching sensory feedback can effectively boost the subjective time compression induced by continuous action. This constitutes a novel finding because no previous studies have successfully demonstrated the possibility of artificially compressing the subjective time experience by enriching sensory feedback during voluntary action.

It is important to note that our study involved at least two task-relevant factors that may have influenced the results. The first factor concerns the temporal cues provided by the auditory feedback. Enriched auditory feedback during the continuous action not only increases the number of matches between predicted and actual outcomes but also provides more temporal cues during the reproduction task. As a result, participants might have been able to more accurately reproduce the duration during the continuous action with multiple auditory feedback compared to single auditory feedback, regardless of the duration of the action. This effect might have conflicted with the observed increase in the compression of subjective time during the continuous action with multiple auditory feedback. The second task-relevant factor is the well-established finding that subjective time compression induced by action tends to decrease as the encoded duration length increases. This is consistent with previous studies using temporal reproduction tasks (Eisler and Eisler, 1992; Sawyer et al., 1994; Noulhiane et al., 2007; Ulbrich et al., 2007; Wittmann et al., 2010), including our own previous work (Ueda and Shimoda, 2021). This pattern aligns with Vierordt’s law (Vierordt, 1868), which proposes that short intervals tend to be over-reproduced and long intervals tend to be under-reproduced when multiple intervals are presented within the same block. In our study, the subjective time compression induced by the continuous action may have conflicted with this factor. Additionally, this trend can be influenced by the method used for duration reproduction (e.g., maintaining a key press throughout the duration versus pressing a key to start and stop the reproduction) (Mioni et al., 2014) and individual reaction times associated with motor responses (Droit-Volet, 2010). Therefore, future studies with more sophisticated experimental designs are needed to disentangle the effects of the continuous action-induced subjective time compression from task-related factors.

The current study has several methodological limitations that should be taken into account. Firstly, our conclusion that enriching sensory feedback can boost subjective time compression induced by continuous action may only apply to auditory feedback. The sensory modality in which information is presented influences perceived duration (Ehrensing and Lhamon, 1966; Hawkes et al., 1977; Penney et al., 1998, 2000; Van Erp and Werkhoven, 2004; Penney and Tourret, 2005; Tomassini et al., 2011). For instance, when auditory and visual durations are mixed in an experimental session, auditory durations tend to be overestimated and visual durations underestimated (Penney et al., 1998, 2000; Penney and Tourret, 2005). Therefore, the effects of enriched sensory feedback may vary depending on the sensory modality used. Further studies employing other types of sensory feedback are necessary to determine whether our findings can be extended to other sensory modalities. Secondly, our findings only pertain to the perception of stimuli above the 1-s time scale, as we used durations of 3 s, 5 s, 7 s, and 9 s. Previous neuroimaging studies have shown that the neural systems involved in temporal processing differ depending on whether the durations are below or above 1-s (Lewis and Miall, 2003; Wiener et al., 2010; Schwartze et al., 2012; Nani et al., 2019). Subcortical structures (e.g., the basal ganglia and cerebellum) are thought to be mainly responsible for the estimation of durations below 1-s, while cortical areas (e.g., the pre-supplementary motor cortex and prefrontal cortex) are thought to be mainly involved in the estimation of durations above 1-s. Therefore, future studies should examine whether our results regarding subjective time compression induced by continuous action can be generalized to stimuli below the 1-s time scale. Thirdly, individual differences may have affected our findings on the relationship between the speed of continuous action and subjective time compression because we did not control the speed of the steering wheel. In our study, participants turned the steering wheel at a self-selected comfortable constant speed. Some studies have reported that the performance of temporal reproduction tasks can be influenced by individual differences, such as impulsivity (Wittmann and Paulus, 2008; Wittmann et al., 2011). Therefore, future studies should control for the speed of continuous action as a within-subjects factor to clarify the relationship between continuous action speed and subjective time compression. Well-controlled replication studies would be valuable for obtaining a deeper understanding of subjective time compression induced by continuous action.

Expanding the applicability of the study’s findings to help individuals modulate their daily time experience through voluntary actions is an interesting research direction. For example, enriched sensory feedback has the potential to enhance various experiences, including driving and using automation technology. Routine driving for work can lead to drivers feeling bored and experiencing subjective time dilation. By appropriately enriching sensory feedback, drivers may avoid dilating their time experience and maintain their sense of agency over their drive, leading to a greater sense of responsibility for their actions on the road. Similarly, automation technology can reduce the number of actions required by operators, potentially leading to increased subjective time dilation and decreased sense of responsibility for task outcomes. To ensure the safe and effective use of automation technology, it’s essential for operators to monitor system performance and be ready to intervene when necessary. Providing appropriate sensory feedback can help operators compress their time experience and maintain their sense of agency over their tasks, ultimately leading to a greater sense of responsibility for task outcomes. Future research should focus on developing effective methods for maximizing subjective time compression. The current study suggests that enriching sensory feedback by increasing the number of matches between predicted and actual action outcomes during voluntary action can enhance the sense of agency and compress the subjective time experience of the action outcome. Multimodal sensory feedback, including visual and tactile feedback in addition to auditory feedback, may increase the number of matches and further enhance subjective time compression. However, it’s important to note that subjective time compression may have a limit. Therefore, future studies should investigate changes in subjective time compression and the sense of agency associated with changes in the amount of sensory feedback from multiple modalities during continuous action, to determine potential plateaus in subjective time compression.

## Conclusion

Our study has demonstrated that enriching auditory feedback can increase subjective time compression during continuous action, which in turn enhances the sense of agency over action outcomes. Importantly, this effect was not caused by a lack of attention toward action outcomes. Our results suggest that increasing the number of matches between predicted and actual outcomes during a voluntary action by providing enriched sensory feedback can effectively compress the subjective time experience of the action outcome. This study is the first to demonstrate the potential of artificially compressing the subjective time experience of daily events through voluntary actions and enriched sensory feedback. Our findings could pave the way for the development of new techniques that allow individuals to control and compress their own time experience. However, further research is necessary to strengthen the validity of our findings and to determine the extent to which these findings can be applied in practical settings.

## Data availability statement

The raw data supporting the conclusions of this article will be made available by the authors, without undue reservation.

## Ethics statement

The studies involving humans were approved by the RIKEN Research Ethics Committee. The studies were conducted in accordance with the local legislation and institutional requirements. Written informed consent for participation in this study was provided by the participants. Written informed consent was obtained from the individual(s) for the publication of any potentially identifiable images or data included in this article.

## Author contributions

SU developed the study concept and performed the data collection and data analysis. SU and SS performed the drafting of the manuscript. SU and SS contributed to the study design. All authors contributed to the article and approved the submitted version.

## Funding

This study received funding from Toyota Motor Corporation. The funder was not involved in the study design, collection, analysis, interpretation of data, the writing of this article or the decision to submit it for publication.

## Conflict of interest

The authors declare that the research was conducted in the absence of any commercial or financial relationships that could be construed as a potential conflict of interest.

## Publisher’s note

All claims expressed in this article are solely those of the authors and do not necessarily represent those of their affiliated organizations, or those of the publisher, the editors and the reviewers. Any product that may be evaluated in this article, or claim that may be made by its manufacturer, is not guaranteed or endorsed by the publisher.

## Footnotes

^1^ https://www.researchgate.net/post/How_compute_a_repeated_measure_power_analysis_in_Gpower
